# Evaluation of a recombinant rhoptry protein 2 enzyme-linked immunoassay for the diagnosis of toxoplasmosis acquired during pregnancy

**DOI:** 10.1590/0074-02760150069

**Published:** 2015-09

**Authors:** Jaqueline Dario Capobiango, Sthefany Pagliari, Aline Kuhn Sbruzzi Pasquali, Beatriz Nino, Fernanda Pinto Ferreira, Thaís Cabral Monica, Nely Norder Tschurtschenthaler, Italmar Teodorico Navarro, João Luis Garcia, Regina Mitsuka-Breganó, Edna Maria Vissoci Reiche

**Affiliations:** 1 Universidade Estadual de Londrina, Centro de Ciências da Saúde, Departamento de Pediatria e Cirurgia Pediátrica, Londrina, PR, Brasil; 2 Universidade Estadual de Londrina, Centro de Ciências Agrárias, Programa de Pós-Graduação em Ciência Animal, Londrina, PR, Brasil; 3 Universidade Estadual de Londrina, Centro de Ciências Agrárias, Departamento de Medicina Veterinária Preventiva, Laboratório de Zoonoses e Saúde Pública, Londrina, PR, Brasil; 4 Secretaria Municipal de Saúde de Cascavel, Cascavel, PR, Brasil; 5 Universidade Estadual de Londrina, Centro de Ciências Agrárias, Departamento de Medicina Veterinária Preventiva, Londrina, PR, Brasil; 6 Universidade Estadual de Londrina, Centro de Ciências Biológicas, Departamento de Ciências Patológicas, Londrina, PR, Brasil; 7 Universidade Estadual de Londrina, Centro de Ciências da Saúde, Departamento de Patologia, Análises Clínicas e Toxicologia, Londrina, PR, Brasil

**Keywords:** toxoplasmosis, pregnancy, rhoptry protein 2, recombinant proteins, Toxoplasma gondii

## Abstract

The aim of this study was to evaluate an enzyme-linked immunoassay with recombinant rhoptry protein 2 (ELISA-rROP2) for its ability to detect*Toxoplasma gondii* ROP2-specific IgG in samples from pregnant women. The study included 236 samples that were divided into groups according to serological screening profiles for toxoplasmosis: unexposed (n = 65), probable acute infection (n = 48), possible acute infection (n = 58) and exposed to the parasite (n = 65). When an indirect immunofluorescence assay for*T. gondii*-specific IgG was considered as a reference test, the ELISA-rROP2 had a sensitivity of 61.8%, specificity of 62.8%, predictive positive value of 76.6% and predictive negative value of 45.4% (p = 0.0002). The ELISA-rROP2 reacted with 62.5% of the samples from pregnant women with probable acute infection and 40% of the samples from pregnant women with previous exposure (p = 0.0180). Seropositivity was observed in 50/57 (87.7%) pregnant women with possible infection. The results underscored that *T. gondii* rROP2 is recognised by specific IgG antibodies in both the acute and chronic phases of toxoplasmosis acquired during pregnancy. However, the sensitivity of the ELISA-rROP2 was higher in the pregnant women with probable and possible acute infections and IgM reactivity.

The clinical diagnosis of toxoplasmosis is difficult because there are few symptoms of the infection. If present, the symptoms are nonspecific and mimic those of other infectious diseases. Serological methods remain an important tool for diagnosing this disease. Prevention and treatment of toxoplasmosis are possible when sensitive and specific methods are used to detect *Toxoplasma gondii *infections (Montoya et al. 2010, Remington et al. 2011). IgM antibodies specific for the pathogen are not accurate markers of the acute phase of infection. In addition, a low IgG avidity index does not always confirm an acute infection due to delayed antibody maturation in some infected individuals (Kotresha & Noordin 2010). Therefore, it is necessary to develop reagents that are able to detect*T. gondii*-specific antibodies for each phase of the disease and to produce these reagents in large quantities for the diagnosis of toxoplasmosis in pregnant women and their children (Wu et al. 2009).

*T. gondii *belongs to the protozoan phylum Apicomplexa, which also includes *Plasmodium *sp., *Cryptosporidium *sp*., Neospora caninum* and *Eimeria *sp*. *These parasites all have an apical complex with secretory organelles, micronemes, rhoptries (ROPs) and dense granules (GRAs) that are involved in host-cell invasion (Hajj et al. 2006). Several genes for these proteins have been cloned and expressed to produce recombinant proteins, such as surface antigens (SAGs), ROPs and GRAs. These proteins are present in *T. gondii*tachyzoite and bradyzoite forms (Kotresha & Noordin 2010). *T. gondii *tachyzoites possess a bundle of eight-12 ROPs (Dubremetz 2007). ROP2, ROP4 and ROP7 are the major protein members that have been identified within the ROP family and are highly specific to *T. gondii*. ROP2 and ROP4 are expressed both in bradyzoites and sporozoites (Hajj et al. 2006).

ROPs contain two sets of proteins that are localised either in the neck, which are called ROP neck proteins and are involved in initial steps of invasion or in the posterior bulb, which are called ROP bulb proteins and are involved in later stages of invasion (Bradley et al. 2005, Dubremetz 2007). ROP2 is involved in the formation of parasitophorous membranes during parasite invasion (Dubremetz 2007, Besteiro et al. 2011).

Several studies have used ROP2 to diagnose toxoplasmosis at different stages of infection in pregnant women (Martin et al. 1998, Macre et al. 2009). The aim of the present study was to evaluate an indirect enzyme-linked immunoassay using recombinant *T. gondii *ROP2 (ELISA-rROP2) to detect ROP2-specific IgG antibodies in serum samples from pregnant women. We also compared the ELISA-rROP2 results with those from a conventional serological method for toxoplasmosis diagnosis.

## SUBJECTS, MATERIALS AND METHODS

*Subjects and serum samples *- The study included 236 serum samples from pregnant women who were evaluated during routine prenatal screening in the Surveillance Health Program for Gestational and Congenital Toxoplasmosis (Lopes-Mori et al. 2011). Samples were obtained from two cities, Londrina and Cascavel, of the state of Paraná, South Brazil, and were stored at -80°C. This study was approved by the Institutional Research Ethical Committee of the State University of Londrina (protocol CEP 043/2011).

The sample size was determined based on an expected proportion of 50% infected pregnant women, with a 95% confidence interval (CI) and a total range of 0.25 for the CI. Sixty-one samples were required for each group that was to be analysed. Because the total extent of the CI was calculated to be 0.30, 43 samples were required for each group (Hulley et al. 2006).

*Sample groups - *The 236 samples were divided into groups according to conventional serological test results. The groups were as follows: group A contained 65 samples from unexposed pregnant women who had nonreactive IgG and IgM anti-*T. gondii*; group B contained 48 samples from pregnant women with probable acute infection and both seroreactivity to IgG and IgM anti-*T. gondii* and low or intermediate IgG avidity; group C contained 58 samples from pregnant women with a possible acute infection and both seroreactivity to IgG and IgM anti-*T. gondii* and high IgG avidity; group I contained 65 samples from pregnant women who had previously been exposed to the parasite and were thus reactive to IgG anti-*T. gondii* and nonreactive to IgM anti-*T. gondii.* The groups were divided based on the case definitions for *T. gondii* infections in pregnant women according to the recommendations of the Ministry of Health of Brazil (MS/SAS/DAPES 2012).

*Conventional serological tests for toxoplasmosis diagnosis -*Prenatal serum samples were tested for anti-*T. gondii *IgM and IgG. Anti-*T. gondii *IgM was detected using a chemiluminescence (CML) immunoassay for microparticles (Architect^®^; System Abbott, Germany) and the samples were characterised as either reactive or nonreactive. Anti-*T. gondii *IgG was detected by indirect immunofluorescence (IFI) using parasite-fixed slides (Imuno-COM^®^; WAMA Diagnóstica, Brazil) and an initial serum dilution of 1:16 (Camargo 1973). Serum values < 1:16 were considered susceptible and serum values ≥ 1:16 were considered exposed or immune to the parasite. In the same samples, anti-*T. gondii *IgG was quantitated using CML (Architect^®^) and expressed as IU/mL. In samples containing both anti-*T. gondii *IgG and IgM, the avidity of the IgG was determined using CML (Architect^®^) and expressed as low, intermediate, or high.

*rROP2 antigen - *The rROP2 antigen was prepared as previously described (Igarashi et al. 2008). Briefly, an 1,103-bp fragment (nucleotides 1022-2125, encoding amino acids 196-561) (Martin et al. 1998, Nigro et al. 2001) of the *ROP2* gene (GenBank accession Z36906) was expressed in *Escherichia coli. *A ~47 kDa rROP2 protein was obtained and evaluated using sodium dodecyl sulfate polyacrylamide gel electrophoresis. The protein concentration was determined using the Lowry method (Lowry et al. 1951) and adjusted to 2.5 µg/mL.

*ELISA-rROP2 for detecting anti-T. gondii IgG -* To detect IgG antibodies against *T. gondii, *an ELISA with rROP2 was performed according to a previously described method (Igarashi et al. 2008), with some modifications. Optimal ELISA conditions were determined using checkerboard titrations with different dilutions of serum samples, rROP2 and peroxidase-conjugated goat anti-human IgG. Briefly, microplates (Nunc-Immuno Plate; MaxiSorp, Denmark) were coated with rROP2 antigen (2.5 µg/mL) that was diluted in 0.1 M carbonate buffer (pH 9.6) and were incubated overnight at 4ºC. The plates were washed three times with phosphate-buffered saline/Tween (PBS-T) (0.05% Tween 20, pH 7.4). Free sites were blocked with 8% nonfat milk in PBS-T and incubated for 1 h at 37ºC. The microplates were washed again, as described above. Serum from all of the groups and 100 µL of positive and negative controls were used in the microplates. All samples were diluted 1:200 in PBS-T 5% nonfat milk and incubated for 1 h at 37ºC. After being washed, 100 µL of peroxidase goat anti-human IgG diluted at 1:20,000 in PBS-T 5% nonfat milk was added to each well. After an 1-h incubation at 37ºC, the microplates were washed three times and developed by adding 100 µL of a chromogenic substrate (40 mg ortho-phenilenediamine/100 mL of 0.1 M phosphate citrate buffer, pH 6.0 and 40 µL of H_2_O_2_). The colour reactions were stopped with 50 µL of 1 N HCl. The optical density (OD) of each sample at 450 nm and 620 nm was determined using a microplate reader (Spectra II Microplate Reader; Tecan Group, Switzerland). Positive and negative control sera were included on each microplate, all samples were run in duplicate and the results were reported as the mean OD values. Cut-off values were expressed as the mean OD of the negative sera (6 wells per microplate) plus two standard deviations (Gatkowska et al. 2010, Pagliari 2013). A sample was considered positive when the sample/cut-off ratio was ≥ 1.0 (Gatkowska et al. 2010).

*Determination of ELISA-rROP2 specificity -* Serum samples from 21 patients with unrelated diseases were assayed using the ELISA-rROP2 to evaluate the specificity of the rROP2 antigen. The patients were previously diagnosed with Chagas disease (n = 4), syphilis (n = 2), paracoccidioidomycosis (n = 2), human immunodeficiency virus type 1 infection (n = 4) and leishmaniasis (n = 1), and some demonstrated seropositivity for antinuclear antibodies (n = 4) and double-stranded DNA antibodies (n = 4). None of the samples were positive for anti-*T. gondii*IgG or IgM by IFI and CML, which are the conventional serological methods used in routine screening for toxoplasmosis.

*Statistical analysis - *Statistical analyses were performed using GraphPad Prism (GraphPad Software Inc, USA) and the SPSS 15.0 software (IBM Corporation, USA). Categorical variables were expressed as an absolute number and percentage and were analysed using a chi-square or Fisher’s exact test. Continuous variables were expressed as the median and 25% and 75% interquartile ranges (IQRs). For continuous variables with three groups, such as those observed when comparing the antibody levels between sample groups, a Kruskal-Wallis test was used. Sensitivity, specificity, positive predictive value (PPV) and negative predictive value (NPV) were determined for all serological methods performed. To assess the concordance between the results of the ELISA-rROP2, IFI and CML methods, the kappa (k) index was determined. A p value < 0.05 was considered statistically significant.

## RESULTS

This study included 257 serum samples from 236 pregnant women who ranged in age from 12-48 years [median: 26 years (25% and 75% IQRs of 21 and 32 years, respectively)]. In addition, 21 samples were characterised as nonreactive for anti-*T. gondii*IgG by serological methods, but were reactive for other unrelated diseases, and they showed no IgG reactivity in the ELISA-rROP2 and were thus added to group A for the statistical analysis.

When the IFI method was used as the reference test for detecting anti-*T. gondii *IgG in the serum samples, the CML assay had a higher sensitivity, specificity, PPV, NPV and k compared with the ELISA-rROP2 (Table I). When the CML was used as the reference test, the IFI method had a higher sensitivity, specificity, PPV, NPV and k than did the ELISA-rROP2 (Table II).

**TABLE I:** Sensitivity, specificity, positive predictive value (PPV), negative predictive value (NPV) and kappa (k) index for the detection of IgG anti-*Toxoplasma gondii* using the chemiluminescence (CML) method and the indirect enzyme-linked immunoassay using recombinant *T. gondii *rhoptry 2 (ELISA-rROP2) as the antigen compared with indirect immunofluorescence (IFI) as the reference test for the serodiagnosis of toxoplasmosis in the serum samples from pregnant women

IgG anti-*T. gondii* (IFI)						p
IgG anti-*T. gondii*	Sensitivity (%) (95% CI)	Specificity (%) (95% CI)	PPV (%) (95% CI)	NPV (%) (95% CI)	k	
CML	97.7 (94.1-99.4)	98.8 (93.7-99.8)	99.4 (96.7-100.0)	95.5 (88.9-98.8)	0.957	< 0.0001^*a*^
ELISA-rROP2	61.8 (54.0-69.1)	62.8 (51.7-73.0)	76.6 (68.7-83.4)	45.4 (36.2-54.8)	0.224	0.0002^*b*^

*a*: Fisher exact test; *b*: chi-square test; CI: confidence interval; total samples: 256 (1 sample was excluded from group C).

**TABLE II:** Sensitivity, specificity, positive predictive value (PPV), negative predictive value (NPV) and kappa (k) index for the detection of IgG anti-*Toxoplasma gondii* using indirect immunofluorescence (IFI) and the indirect enzyme-linked immunoassay using recombinant *T. gondii *rhoptry 2 (ELISA-rROP2) as antigen compared with the chemiluminescence (CML) method as the reference test for the serodiagnosis of toxoplasmosis in the serum samples from pregnant women

IgG anti-*T. gondii* (CML)						p
IgG anti-*T. gondii*	Sensitivity (%) (95% CI)	Specificity (%) (95% CI)	PPV (%) (95% CI)	NPV (%) (95% CI)	k	
IFI	99.4 (96.7-100.0)	100 (95.8-100.0)	100 (97.8-100.0)	98.8 (93.7-99.8)	0.991	< 0.0001^*a*^
ELISA-rROP2	62.3 (54.5-69.7)	62.4 (51.2-72.6)	76.5 (68.4-83.3)	45.7 (36.4-55.2)	0.226	0.0002^*b*^

*a*: Fisher exact test; *b*: chi-square test; CI: confidence interval; total samples: 252 (5 samples were excluded: 1 from group A, 2 from group B and 2 from group I).

The sensitivity, specificity, PPV, NPV and k of the ELISA-rROP2 were significantly lower than those of the CML and IFI assays. This applied to the samples from pregnant women with probable acute infection (group B), with possible acute infection (group C) and previously exposed (group I), as shown in Tables III, IV, respectively.

**TABLE III:** Sensitivity, specificity, positive predictive value (PPV), negative predictive value (NPV) and kappa (k) index of IgG anti-*Toxoplasma gondii* obtained using the indirect enzyme-linked immunoassay using recombinant *T. gondii*rhoptry 2 as antigen compared with the chemiluminescence (CML) method as the reference test in serum samples from the pregnant women with probable acute infection and low avidity IgG anti-*T. gondii* (group B), possible acute infection and high avidity (group C) IgG anti-*T. gondii* and previously exposed to*T. gondii* (group I)

Groups	CML (n) R/total NR/total		Sensitivity (%) (95% CI)	Specificity (%) (95% CI)	PPV (%) (95% CI))	NPV (%) (95% CI)	k	p^*a*^
B	28/46	32/85	60.9 (45.4-74.9)	62.4 (51.2-72.6)	46.7 (33.7-60.0)	74.7 (62.9-84.2)	0.217	0.0109
C	50/58	32/85	86.2 (74.6-93.9)	62.4 (51.2-72.6)	61 (49.6-71.6)	86.9 (75.8-94.2)	0.456	< 0.0001
I	26/63	32/85	41.3 (29.0-54.4)	62.4 (51.2-72.6)	44.8 (31.7-58.5)	58.9 (48.0-69.2)	0.045	0.6553

*a*: chi-square test; CI: confidence interval; NR: nonreactive samples; R: reactive samples; total samples: 252.

**TABLE IV:** Evaluation of the sensitivity, specificity, positive predictive value (PPV), negative predictive value (NPV) and kappa (k) index of IgG anti-*Toxoplasma gondii* using the indirect enzyme-linked immunoassay using recombinant *T. gondii *rhoptry 2 compared with the indirect immunofluorescence (IFI) method as the reference test in the serum samples from the pregnant women with probable acute infection with low avidity (group B) and possible acute infection with high avidity (group C) of IgG anti-*T. gondii* and previously exposed to *T. gondii*(group I)

Groups	IFI (n) R/total NR/total		Sensitivity (%) (95% CI)	Specificity (%) (95% CI)	PPV (%) (95% CI)	NPV (%) (95% CI)	k	p^*a*^
B	30/48	32/86	62.5 (47.4-76.1)	62.8 (51.7-73.0)	48.4 (35.5-61.4)	75 (63.4-84.5)	0.238	0.0049
C	49/57	32/86	86 (74.2-93.7)	62.8 (51.7-73.0)	60.5 (49.0-71.2)	87.1 (76.2-94.3)	0.455	< 0.0001
I	26/65	32/86	40 (28.0-52.9)	62.8 (51.7-73.0)	44.8 (31.7-58.5)	58.1 (47.4-68.2)	0.028	0.7270

*a*: chi-square test; CI: confidence interval; NR: nonreactive samples; R: reactive samples; total samples: 256.

Using the IFI method as the reference test, 30/48 (62.5%) women with a probable acute infection (group B) and 26/65 (40%) previously exposed women (group I) had positive ELISA-rROP2 results (p = 0.0180). The frequency of IgG reactivity that was observed when using the ELISA-rROP2 to examine the other groups of pregnant women was also evaluated. Seropositivity was observed in 32/65 (49.2%) of the unexposed pregnant women (group A) and in 50/57 (87.7%) of those with a possible acute infection (group C).

The levels of anti-*T. gondii *IgG determined by the ELISA-rROP2 were significantly different between groups A (unexposed) and C (possible acute infection) (p < 0.0001), between groups B (probable acute infection) and C (p < 0.0001) and between groups C and I (previously exposed) (p < 0.0001) (Figure).

## DISCUSSION

Recombinant proteins can be useful for the diagnosis of toxoplasmosis because the isolation of *T. gondii* from cultures or animal models is expensive, laborious, time-consuming and potentially hazardous. The *T. gondii*protein ROP2 is present in tachyzoites, bradyzoites and sporozoites (Van-Gelder et al. 1993) and is very antigenic because it strongly induces humoral and cellular immune responses in the *T. gondii-*infected host. ROP2 has been cloned and expressed in vitro and may thus be used for the diagnosis of and vaccination against toxoplasmosis. Indeed, IgA, IgM and IgG anti-*T. gondii* antibodies in human toxoplasmosis can be detected by using rROP2 expressed in *E. coli* (Chang et al. 2011, Yan et al. 2012). An adequate selection of recombinant antigens may also be used in serological tests to differentiate between recently and previously acquired infections. Properly defining the stage of a toxoplasmosis infection in pregnant women is very important for guiding the medical treatment (Martin et al. 1998, Buffolano et al. 2005, Wu et al. 2009).

**Figure:**
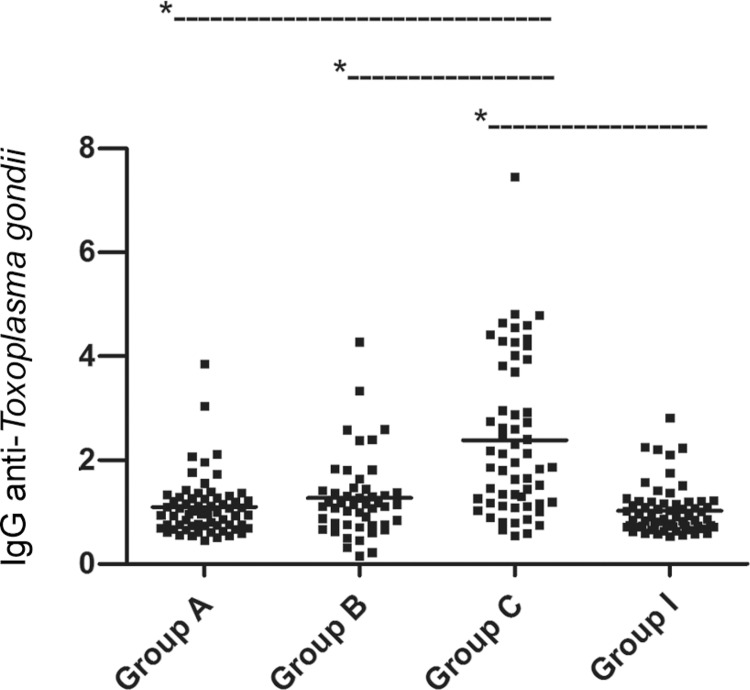
Levels of IgG anti-*T. gondii* antibodies obtained using the indirect enzyme-linked immunoassay using recombinant*T. gondii *rhoptry 2 as the antigen in the samples of pregnant women nonexposed (group A), with probable acute infection (group B), with a possible acute infection (group C) and previously exposed to *T. gondii* (group I). The values are expressed as ELISA index ≥ 1, which were obtained using the mean optical density (OD) of sample/mean OD for the negative controls plus 2 standard deviation. The bars represent the median of the titres of each group (Kruskal-Wallis test with Dunn’s post-test). Asterisk means p < 0.0001. For comparison with other groups, the difference was not significant (p > 0.05).

In this study, *E. coli*-expressed rROP2 was used in an ELISA to detect anti-*T*. *gondii *IgG antibodies in the serum of pregnant women. This assay was able to detect reactivity to IgG in 89% of the serum samples from patients with acute toxoplasmosis and in 78% of the samples from patients with a chronic infection (Van-Gelder et al. 1993). However, our study did not evaluate IgM anti-*T. gondii* with the ELISA-rROP2.

We found that the ELISA-rROP2 had the highest positivity in the samples from pregnant women with toxoplasmosis and a possible acute infection (group C) followed by groups B and I. These results demonstrate that the ELISA-rROP2 can detect both acute and chronic infections during pregnancy. The results are also consistent with previous studies indicating that rROP2 induces a humoral immune response that produces IgM, IgA and IgG antibodies. IgM and IgA are made during the acute phase of a*T*. *gondii *infection, whereas IgG is made during both acute and chronic stages (Van-Gelder et al. 1993, Martin et al. 1998). Anti-*T*. *gondii *IgM was detected in 62.1% of the samples from Brazilians and Argentines using an IgM-immunosorbent agglutination assay with ROP2 as the antigen (Martin et al. 1998). Moreover, these authors reported that anti-*T*.*gondii* IgG was detected in 91% of the samples by ROP2. ROP2 reacted with 98% of samples from patients with an acute infection and 83% of samples from patients with a chronic infection, which suggests that anti-ROP2 antibodies are present in both chronic and acute toxoplasmosis (Martin et al. 1998).

Macre et al. (2009) used the ELISA-rROP2 to evaluate the anti-*T. gondii* IgG levels in pregnant Brazilian women with acute toxoplasmosis. Using a conventional ELISA as the reference test, these authors obtained excellent concordance between the conventional and ELISA-rROP2 methods. The ELISA-rROP2 had a sensitivity of 87%, specificity of 88%, PPV of 98% and NPV of 43%. However, when the ELISA-rROP2 was used to assess IgG avidity, the sensitivity and accuracy were inferior. The authors also reported that rROP2 exhibited good stability for an immunoblotting assay when stored at -70°C (Macre et al. 2009).

In a previous study, we demonstrated a low sensitivity and specificity for the ELISA-rROP2 when detecting IgG in samples from individuals with acute and chronic toxoplasmosis. The sensitivity increased when detecting IgM anti-rROP2 during an acute infection (Pagliari 2013).

The ELISA-rROP2 did not react with serum samples from patients with other diseases, consistent with a previous study (Martin et al. 1998), in which the ELISA-rROP2 did not react with samples of patients who were seronegative for toxoplasmosis and seropositive for other diseases. However, in the present study, the samples in group A had low specificity, which can be partly explained by the inability of the ELISA-rROP2 to recognise some of the antigens used in commercial tests because of the genetic diversity of the host immune response. These samples in group A could thus be considered false negatives when using conventional tests, such as IFI and CML. Other explanations include a recent infection with *T. gondii *around the time of the sample collection (Sun et al. 2013) and the potential lot-to-lot variability of the rROP2 protein. All of the samples from patients with other diseases were evaluated on the same day using a unique lot of rROP2.

Several conventional serologic tests that are commercially available for diagnosing*T. gondii* infections, such as IFI, utilise whole extracts of tachyzoites, which contain many proteins that can influence the results of a test. Some authors have reported that tests using whole extracts have pseudopositivity concordance, with higher positivity when compared to western blotting (WB) (Wu et al. 2009).

Using WB on samples from patients with toxoplasmosis, amebiasis, cysticercosis, filariasis, malaria or toxocariasis, the sensitivity and specificity for diagnosing toxoplasmosis with rROP2 was 90% and 95%, respectively (Chang et al. 2011). In that study, rROP2 was better at detecting IgG (91.7%) than IgM (80%) (Chang et al. 2011). We did not perform WB, which is likely to have a different sensitivity and specificity compared with the ELISA-rROP2.

Using recombinant antigens to detect anti-*T. gondii *IgG in an ELISA is more sensitive than using whole parasites, such as *T. gondii*taquizoites of RH and ME49 strains. However, due to the genetic diversity of the*T. gondii* isolates, a method that can detect most of the different *T. gondii*-specific antibodies is needed (Sun et al. 2013).

The ability of 11 different *T. gondii *recombinant antigens to detect IgG and IgM using an ELISA was previously evaluated, with rROP2 reacting with 58% of serum samples from individuals with chronic toxoplasmosis and 80% of those from individuals with potential acute toxoplasmosis (Aubert et al. 2000). Moreover, using the combination of three recombinant antigens (p29, p30 and p35) to detect IgG and three recombinant antigens (p29, p35 and p66) to detect IgM improved the sensitivity from 93.1-98.4% (Aubert et al. 2000). Other rROPs have been evaluated, such as rROP8, which had a specificity of 94% and sensitivity of 90% for early acute toxoplasmosis and sensitivities of 92% and 82% for acute and chronic toxoplasmosis, respectively (Sonaimuthu et al. 2014). Liu et al. (2012)demonstrated that 100% of the serum samples that contained anti-*T. gondii*IgM, but not IgG showed positive IgM. However, none of them showed positive IgG when rROP2 was used as an antigen in pregnant women. These results indicate that rROP2 can be used to capture anti-*T. gondii *IgM antibodies. In the present study, the ELISA-rROP2 was not used to detect IgM. However, measuring IgM antibodies in the ELISA-rROP2 may improve its performance in diagnosing acute toxoplasmosis in pregnant women.

SAG1, which is a major *T. gondii *SAG, is used in some commercially available tests because it exhibits high antigenicity and immunogenicity (Gatkowska et al. 2010).Therefore, a combination of ROP proteins with other *T. gondii* antigens, such as SAG1, may improve the assay’s performance. Using only a single antigen in an ELISA can result in false negatives because some infected individuals may not produce antibodies against epitopes found on the recombinant antigen. Hence, a combination of different antigens in the same test is likely to be more sensitive than using a single antigen (Van-Gelder et al. 1993, Aubert et al. 2000, Gatkowska et al. 2010).

Interestingly, Van-Gelder et al. (1993)explained that their recombinant antigen was unable to react with antibodies in approximately 10% of infected individuals because the IgG response to ROP2 is delayed. This finding suggests that ROP2 antibodies are increased in the chronic stage of toxoplasmosis. In the very early stage of infection, antibodies against the ROP2 antigen are undetectable. In the present study, the ELISA-rROP2 reacted with the same specificity to samples from acute infection and samples from chronic infection. However, similar to Liu et al. (2012), we detected a higher sensitivity and NPV in the samples known to contain anti-*T. gondii *IgM in the groups with probable acute infection (B) and possible acute infection (C) than in the other groups (A and I).

Moreover, the false negatives could be due to variations in the procedure used to obtain the rROP2, which can compromise the stability and conformational structure of the epitopes and prevent their recognition by specific antibodies (Gatkowska et al. 2010).

The false positives in the samples from susceptible pregnant women (group A) may be explained by the presence of bacterial proteins contaminating the rROP2 protein preparation. This possibility underscores the need to increase the purity of the recombinant protein by improving the purification procedure. As reported by Pagliari (2013), using a more purified antigen may reduce the noise level of the negative controls in the ELISA-rROP2.

Expressing rROP2 as a recombinant protein in *E. coli* is not easy. rROP2 is expressed by *E. coli *at low levels with low solubility and a high degree of protein degradation (Van-Gelder et al. 1993). A previous study described these problems, although the investigators achieved high levels of rROP2 expression when the bacteria were grown in broth (Nigro et al. 2001). We had some difficulties in achieving adequate expression and preventing degradation of rROP2, which were likely due to the high sensitivity of rROP2 to bacterial proteases. The rROP2 protein used in the present study was always expressed at a low level and found to be insoluble when purified. Degradation was observed in the expression and purification stages, as well as during storage of the protein (Pagliari 2013), which may have contributed to the decreased reactivity of the ELISA-rROP2. Previously, a yeast expression system was used to easily and quickly produce recombinant proteins on a large scale in inexpensive media (Chang et al. 2011).

Taken together, our results confirm that rROP2 can be recognised by antibodies produced in both the acute and chronic phases of *T. gondii*infection. In addition, the ELISA-rROP2’s sensitivity was higher in the samples from pregnant women with probable or possible acute infections with reactivity for IgM. Therefore, the combination of rROP2 with other recombinant antigens should be investigated to differentiate the phases of toxoplasmosis in pregnant women.
